# ASFV infection induces lipid metabolic disturbances and promotes viral replication

**DOI:** 10.3389/fmicb.2024.1532678

**Published:** 2025-01-07

**Authors:** Xuefei Chu, Shengqiang Ge, Yingchao Li, Qin Zhang, Xinyu Cui, Yuanyuan Zuo, Ruihong Li, Hongtao Sun, Lei Yin, Zhenzhong Wang, Jinming Li, Yihong Xiao, Zhiliang Wang

**Affiliations:** ^1^College of Veterinary Medicine, Shandong Agricultural University, Tai'an, Shandong, China; ^2^China Animal Health and Epidemiology Center, Qingdao, China; ^3^Qingdao Key Laboratory of Modern Bioengineering and Animal Disease Research, Qingdao, China; ^4^Key Laboratory of Animal Biosafety Risk Warning Prevention and Control (South China), Ministry of Agriculture and Rural Affairs, Qingdao, Shandong, China; ^5^College of Veterinary Medicine, Qingdao Agricultural University, Qingdao, Shandong, China

**Keywords:** African swine fever virus, lipid metabolism, cholesterol, lipid synthesis, transcriptomic analysis

## Abstract

**Introduction:**

African swine fever is a highly transmissible and lethal infectious disease caused by the African swine fever virus (ASFV), which has considerably impacted the global swine industry. Lipid metabolism plays a vital role in sustaining lipid and energy homeostasis within cells and influences the viral life cycle.

**Methods and results:**

In this study, we found that ASFV infection disrupts lipid metabolism in the host. Transcriptomic analysis of cells infected with ASFV revealed that the levels of lipid metabolism significantly changed as the duration of the infection progressed. The intracellular cholesterol levels of the host exhibited a pattern similar to the viral growth curve during the course of infection. Notably, increased cholesterol levels promoted ASFV replication in host cells, whereas inhibition of the cholesterol biosynthesis pathway markedly reduced intracellular ASFV replication.

**Discussion:**

The findings of this study showed that ASFV led to lipid metabolism disturbances to facilitate its replication, which is useful for revealing the mechanism underlying ASFV infection.

## 1 Introduction

The unique structure and composition of virus particles dictate their capacity to enter host cells through intricate interactions with cellular components. While certain viruses can penetrate the plasma membrane and enter the cytoplasm directly, the majority rely on endocytic uptake. Viruses are subsequently delivered to endosomes and other intracellular organelles through vesicular transport in the cytoplasm (Mercer et al., 2010). The internalization of viruses may involve clathrin-mediated endocytosis (CME), macropinocytosis, vesicular-and lipid raft-mediated endocytosis, or various other poorly characterized mechanisms (Mercer et al., 2010). Viruses often exploit cellular endocytosis to facilitate their entry into host cells, thereby evading immune detection. Endocytic vesicles transport invading viruses from the periphery to the perinuclear region of the host cell, allowing viruses to bypass obstacles associated with cytoplasmic crowding and the microfilament meshwork in the cortex, thereby enhancing intracellular replication (Cuesta-Geijo et al., 2016). Endocytosis serves as a prevalent uptake pathway for nutrients, lipids, and receptors that are frequently exploited by viruses to gain entry into cells (Cuesta-Geijo et al., 2016). Regardless of whether the virus entrying into cells by hijacking the host cell’s endocytosis or via the virus’s intracellular transport processes, the maintenance of adequate cholesterol flux and energy supply is crucial (Goodwin et al., 2015; Coutinho et al., 2018). Most viruses modulate the lipid and glucose metabolism of the host, which is essential for supporting their infection and replication (Thompson et al., 2011). Upon invading host cells, viruses utilize the host’s metabolic systems to support their proliferation, particularly by manipulating the metabolism of glucose (Jin et al., 2010; Gonnella et al., 2013; Huang et al., 2015), glutamine (Newsholme et al., 2003), and fatty acids (Koyuncu et al., 2013). These activities facilitate viral proliferation, construction, and egress (Li et al., 2023).

African swine fever (ASF) is a highly transmissible, acute, lethal infectious disease. African swine fever virus (ASFV) is capable of infecting domestic and wild pigs across all breeds and ages, with morbidity and mortality rates up to 100%. Outbreaks of ASF in either new or endemic regions have significant local socioeconomic consequences. ASFV is a large double-stranded DNA virus that enters host cells through dynamin-, clathrin-, and cholesterol-dependent endocytosis (Hernaez and Alonso, 2010; Hernáez et al., 2010; Galindo et al., 2015; Chen X. et al., 2023). Virus particles are uncoated within minutes at acidic pH upon entering the endosome, and subsequently releasing their genetic material into the cytoplasm, where replication occurs in specialized structures known as virus factories (Cuesta-Geijo et al., 2012; Netherton and Wileman, 2013). Unlike other DNA viruses, such as vaccinia virus or adenovirus 5, cholesterol efflux from endosomes is required for ASFV release and entry into the cytosol. The accumulation of cholesterol in endosomes disrupts fusion, resulting in the retention of virions within endosomes (Cuesta-Geijo et al., 2016). Like other viruses (Mazzon et al., 2013; Greseth and Traktman, 2014; Pant et al., 2021), ASFV has evolved specific proteins, particularly those expressed early, to regulate host cell endocytosis and transport pathways, thereby enhancing self-infection and replication (Gómez-Puertas et al., 1996; Chen et al., 2022). These pathways are intricately linked to lipid synthesis and energy metabolism.

The disruption of lipid metabolism homeostasis is a critical characteristic of viral infections, with viruses utilizing the host lipid machinery to support their replication cycle, while undermining the host immune response (Proto et al., 2021). HSV-1 infection inactivates glutathione peroxidase 4 (GPX4), which leads to increased lipid peroxidation and subsequent inhibition of STING protein transport from the endoplasmic reticulum to the Golgi apparatus (Jia et al., 2020). This disruption hampers the innate immune response, thereby facilitating HSV-1 replication *in vivo*. The internalization of ASFV relies on sufficient cholesterol levels in the cytosolic membrane to enable the fusion of ASFV with the host cell’s endosomal membrane, where sterols are vital for membrane fusion (Bernardes et al., 1998). During the initial phases of ASFV infection, cholesterol flux is crucial for establishing a productive infection. ASFV also alters intracellular cholesterol dynamics by increasing cholesterol cellular uptake and redistributing free cholesterol to sites of viral replication (Cuesta-Geijo et al., 2016).

The metabolic disruptions caused by ASFV infection remain poorly understood, and research on how abnormalities in lipid metabolism influence viral replication during the later stages of infection is limited. In this study, we validated the metabolic state and levels of metabolism in vivo and *in vitro* following ASFV infection. Additionally, the effects of abnormal lipid metabolism on ASFV replication during the late stages of infection were investigated. This study deepens the understanding of the molecular epidemiology of ASF and provides valuable insights for the development of vaccines against ASF.

## 2 Materials and methods

### 2.1 Cells and virus

ASFV virulence strain ASFV China/LN/2018/1 (GenBank accession number OP856591, abbreviated as ASFV-CN2018) were provided by the China Animal Health and Epidemiology Center (CAHEC). The wild boar lung cell line (WSL) were kindly provided by Matthias Lenk from the Friedrich-Loeffler-Institute (FLI), Greifswald-Insel Riems, Germany. WSLs were cultured in Dulbecco’s modified Eagle’s medium (DMEM) containing 10% fetal bovine serum (catalog no. D6429; Sigma-Aldrich) and 1% antibiotics-antimycotics.

### 2.2 Animal experiments

Twelve 8-week-old healthy specific pathogen-free piglets were randomly assigned into two groups (6 piglets inoculated with ASFV China/LN/2018/1 at 50 HAD_50_ per piglet (1 mL, intramuscular); 6 piglets inoculated with PBS). The piglets were monitored daily for clinical signs prior to feeding, including anorexia, lethargy, fever, and emaciation. Serum samples were collected on days 0, 5, and 9 post-infection (dpi) to detect metabolism-related proteins. ASFV infected piglets were euthanized in the moribund stage. The tissue samples from heart, lung, spleen, liver, kidney and lymph nodes (mesenteric lymph node, inguinal lymph node and submandibular lymph node) were collected for inflammatory cytokines and virus load detection.

### 2.3 Real-time PCR analysis

Total RNA was extracted from different tissues and cells in each group, after which the concentration was determined and the RNA was reverse transcribed to cDNA. Gene expression levels were tested by real-time PCR using SYBR Green detection system. The expression levels of these genes were normalized to the *GAPDH*. The final mRNA levels of these genes in this study were normalized using the comparative cycle threshold method. The primers were synthesized by Sangon Biotech (Shanghai, China), and sequences of primers were listed in Table 1.

**Table 1:** Details of constructs and primers.

Constructs	Primers	Sequence (5′ → 3′)
ASFV-CN2018-p72	F	TCTTTGGCGCCTAGCTGTCT
	R	GGCGCAAATATCAACTATGGTTT
	P	TCTCGGATGTGCTTCGT
APOE	F	ATGAGGGTTCTGTGGGTTGC
	R	CTTGGTCAGACAGGGACTGC
RBP-4	F	GGACTATGACACCTACGCCG
	R	GAGTGATCAGCCGGTACTGC
CETP	F	TGCAGTTGGGTTAGCCTGAG
	R	CCTCCCTCCACCCTTACTGT
Lp-PLA2	F	CTGACCTGGCATCTTACGGG
	R	CCCCTTTTCCGAGGGTTCTC
OGT	F	GCCCAGATGATGGCACAAAC
	R	GAGTTCATTGCGAGCACCCT
GLUT1	F	CCAGGTATTTGGCCTGGACT
	R	GGGTCACGTCTGCCGTTC
LDH-A	F	TAAGGAAGAGCATGTTCCCCAC
	R	TGGGGTCCTAAGGAAAAGGC
APOH	F	TGCTGTAGTTGTGCCGTTGA
	R	ATGGGCCAAAGTCCTGTGAG
ABCA7	F	TCAAATACCGCAAAGGTCGC
	R	CAAGGCAGTGTCACCCTTTC
AQP7	F	CTACCCGTGCCTCCAAGATG
	R	CGGAGCCTAGACCAAACACC
BST2	F	AAGGCCCAGTACACCGTTTG
	R	TGAACGCCTTCCTTAGTCGC
C3	F	CTCCCTGTCATCCCACAACC
	R	AAGACTTGCTGGGGAGCATC
DHRS3	F	GAGCGTTGGTAGTGTTCCCT
	R	GCGTTCTGCGAACTCCCT
HSD17B7	F	GCAGCTTGTCATCGGTGTTC
	R	TGGGTCAACAGTCCTTCAGC
SQLE	F	GAGAATATTTGCAGCCAGGCG
	R	AACTTTGCATTGGGCTCTGC
NPC1	F	TCTTTAACCTCGGCGTGTCC
	R	ATTTGGCAAAGGCCAACACC
PLCB2	F	CGAGCAACGGGTCAGCAG
	R	TGCAGCCTGTTTCTCTCTGG
PLTP	F	GCTGCAGGAGGAAGAGCG
	R	CAGCTTCAGCGGAGAGTCAA
PLPP3	F	GCTCCGAAGGCTACATCCAA
	R	TTGTGATCAGACACACGGGA
PID1	F	GGTAAAGTCCCCACTACGGG
	R	AGGTGATGGAGCCACACTTG
RSAD2	F	GGGAGAGGTGGTTCAAGAGC
	R	TGACCACGGCCAATAAGGAC
ZC3H12A	F	ACGGCATCATGGTCTCCAAC
	R	CCCGTAGGTGCATTTCCTCC
siHSD17B7	F	GGACCUAGAUGAAGACACU (dT)(dT)
	R	AGUGUCUUCAUCUAGGUCC (dT)(dT)
siSQLE	F	GAUUCGCUGUAUCAACUAA (dT)(dT)
	R	UUAGUUGAUACAGCGAAUC (dT)(dT)

The WSL cells were counted, then transferred to 24-well plates and cultured in a cell culture incubator for 16 h. The supernatant was discarded and washed once with phosphate-buffered saline (PBS), and serum-free DMEM medium was added. WSLs in 24-well plates were transfected with 400 nM siRNAs against SQLE or HSD17B7 using Lipofectamine RNAiMAX Transfection Reagent (catalog no. 13778030; ThermoFisher Scientific). After incubation for 6 h at 37°C, the cells were infected with ASFV at a multiplicity of infection (MOI) of 1 for 24 h. Subsequently, the cells were subjected to RNA extraction by RNAiso Plus (catalog no. 9109; TaKaRa) according to the manufacturer’s instructions. The siRNA knockdown efficiency of the target genes was assessed by RT-qPCR. The siRNAs were synthesized by Tsingke Biotechnology (China). The primers were synthesized by Tsingke Biotech (Beijing, China), and sequences of primers were listed in Table 1.

### 2.4 Transcriptome analysis

The WSL cells were counted, then transferred to 6-well plates and cultured in a cell culture incubator for 16 h. The supernatant was discarded and washed once with phosphate-buffered saline (PBS), the cells were infected with ASFV at 1MOI for 5, 9, 16, 24 h. Total RNA was extracted from each group, and the construction of the RNA-seq library and sequencing of the libraries was performed by a commercial service. Bioinformatic analysis was performed using the OECloud tools^1^.

### 2.5 Virus growth curve

WSL cells were seeded into 24-well plates and infected with 1MOI of ASFV for 2 h, washed once with PBS, and then cultured in fresh medium. The viral genome copies were detected at 12, 24, 36, 48, 60, 72, 84, and 96 h post-infection (hpi). Take 200 μL sample of virus culture supernatant from each well for viral nucleic acid extraction. Then, viral genome copies of ASFV were quantified by qPCR, and the one-step growth curve was plotted according to viral genome copies.

### 2.6 Quantification of cholesterol contents

Cells were collected into the centrifuge tube, and discard the supernatant after centrifugation; add 1 mL anhydrous ethanol to ultrasonically disrupt the cells in an ice bath for 5 min (power 20%, ultrasonic 3 s, interval 7 s, repeat 30 times). Centrifuge at 8,000 g for 10 min at 4°C. Use supernatant for assay, and place it on ice to be tested. The amount of cholesterol in the samples was measured using the micro total cholesterol assay kit (Abbkine Scientific Co., Ltd) according to the manufacturer’s instructions. The cholesterol level was measured using an SuPerMax-3100 Microplate Reader measurement system (Shanghai Shanpu Biotechnology Co., Ltd). The results were normalized by the total protein concentration of each sample.

### 2.7 Nile red staining

WSL cells were seeded into 12-well culture plates (2 × 10^5^ cells/plate). When cells reached 80–90% confluence, and infected with ASFV at 1MOI or 3MOI for 2 h. Washed once with PBS, and cultured in a fresh medium. WSL cells were fixed in 4% paraformaldehyde for 30 min at 24 hpi. Next, the cells were incubated with Nile red solution for 10 min at 37°C and washed with PBS three times. Then, cell nuclei were stained with DAPI (Abcam Plc) for 10 min at 37°C and observed under a fluorescent microscope.

### 2.8 Statistical analysis

All experiments were performed independently at least three times. A statistical analysis was performed using Student’s *t*-test (*, *p* < 0.05; **, *p* < 0.01; ***, *p* < 0.001; NS indicates no significance). All statistical analyses were completed using GraphPad Prism 8.0 software.

### 2.9 Policies and publication ethics

All ASFV-related experiments were carried out in the BSL-3 laboratory, and the animals were handled in strict accordance with good animal practices according to the Animal Ethics Procedures and Guidelines of the People’s Republic of China. The study was approved by the Animal Welfare Committee of the China Animal Health and Epidemiology Center (Approval Number: DWFL-2023-01).

## 3 Results

### 3.1 ASFV infection disrupts glucose and lipid metabolism in pigs

**Figure 1:**
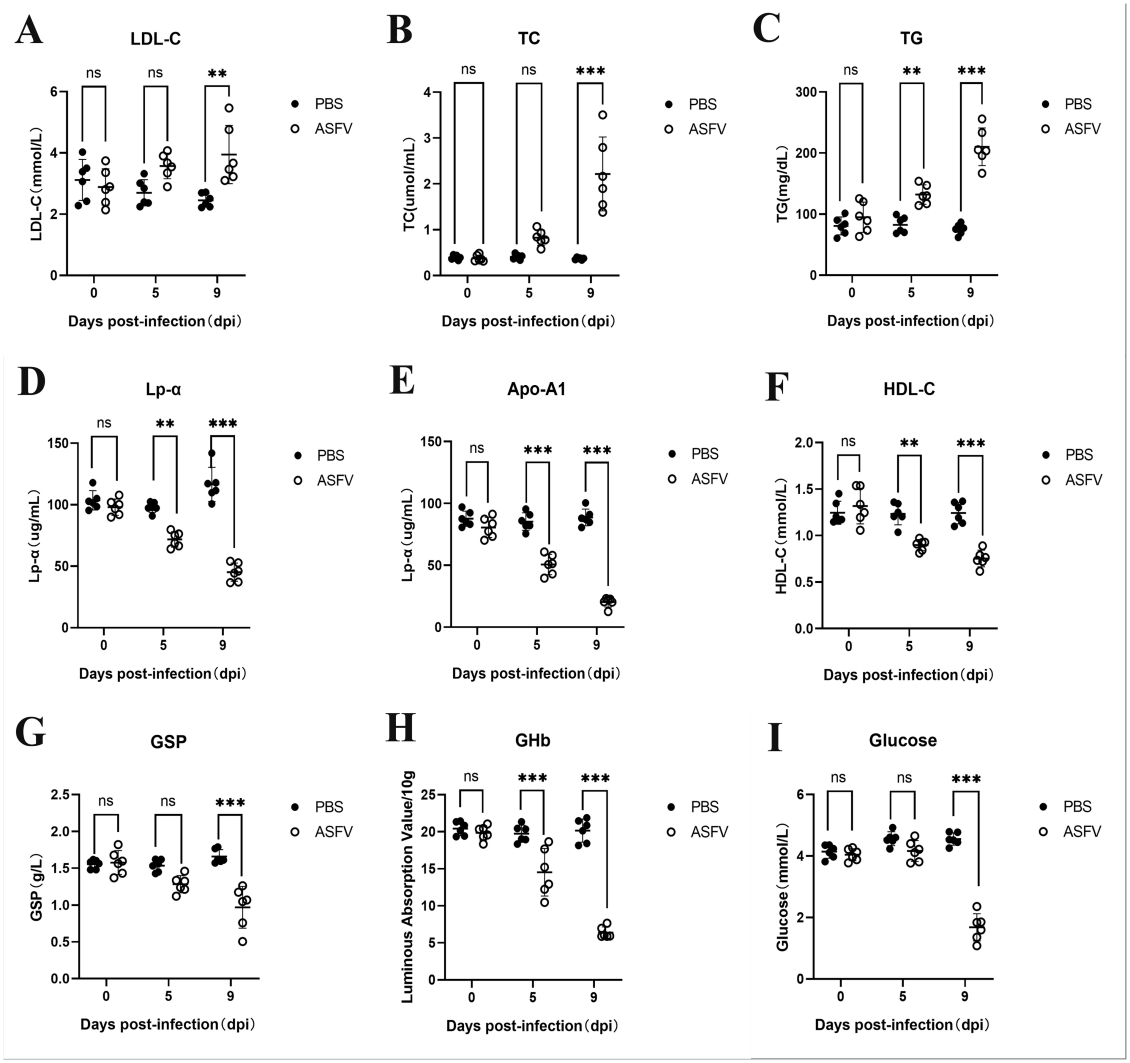
ASFV infection disrupts glucose and lipid metabolism in pigs. **(A)** Pig serum was collected at various time points following ASFV infection to assess changes in the expression of LDL-C. Mock-infected control group: PBS label (black dots); Experimental group: ASFV label (White dots). **(B)** Levels of TC; **(C)** Levels of TG; **(D)** Levels of Lp-*α*; **(E)** Levels of Apo-A1; **(F)** Levels of HDL-C; **(G)** Levels of GSP; **(H)** Levels of GHb; **(I)** Levels of Glucose.

To investigate whether ASFV infection leads to alterations in glucose and lipid metabolism in pigs, serum samples were collected from six pigs in both the experimental and control groups on days 0, 5, and 9 post-inoculation. Metabolism-related proteins were assessed via biochemical assay kits. The results revealed that in the serum from the experimental group, the levels of LDL-C, TC, and TG significantly increased, reflecting a notable increasing trend as the infection progressed (Figures 1A–C). In contrast, the levels of HDL-C, Lp-*α*, and Apo-A1 significantly decreased with increasing infection time (Figures 1D–F). Furthermore, in the ASFV-infected group, the levels of GSP, GHb, and glucose markedly decreased as the infection progressed (Figures 1G–I). These results indicate that ASFV infection leads to substantial disturbances in lipid and glucose metabolism in pigs.

### 3.2 ASFV infection modulates the metabolic levels in the host

**Figure 2:**
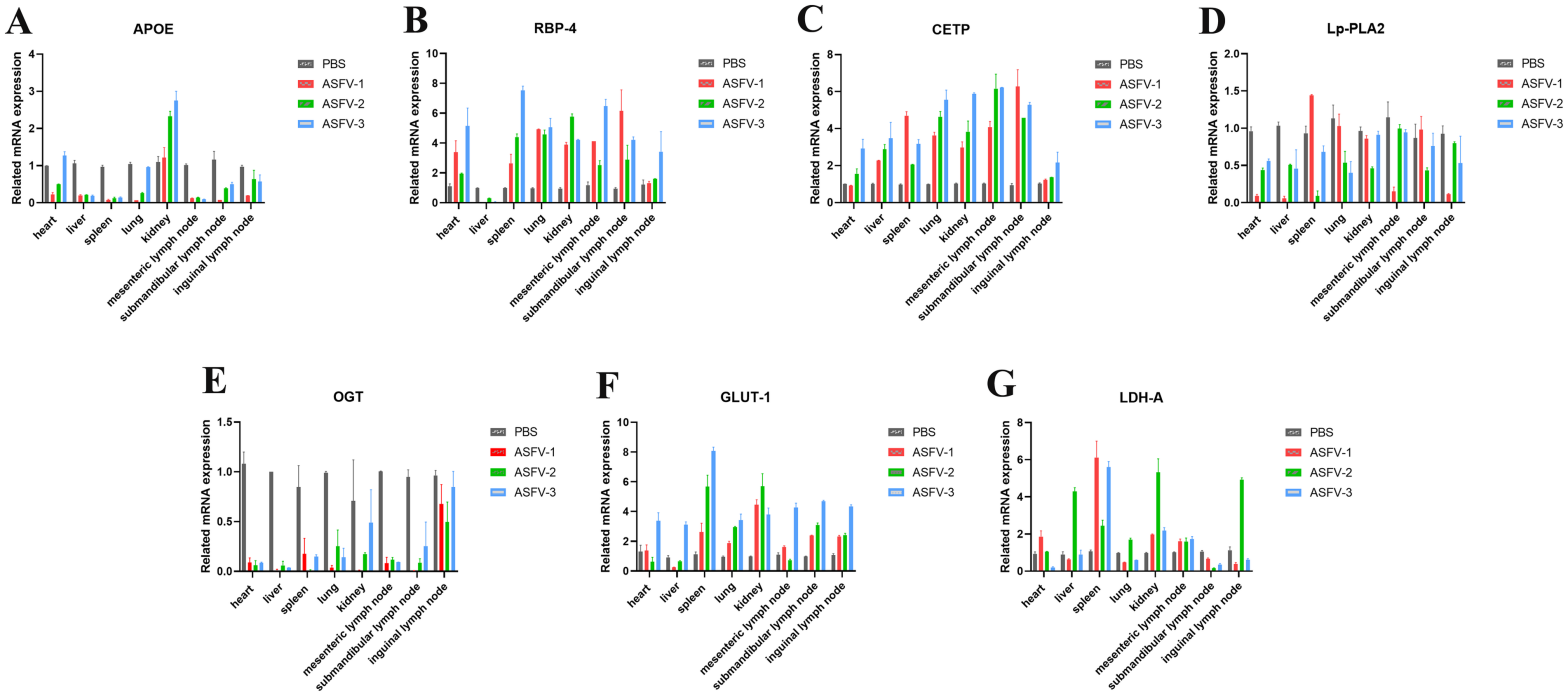
ASFV infection modulates the metabolic levels in the host. **(A)** Collection of different tissues from the pigs following ASFV infection to detect expression levels of APOE. Mock-infected control group: PBS label (gray bars); Experimental group: ASFV-1 (red bars), ASFV-2 (green bars), ASFV-3 (blue bars), represent three random pigs by ASFV-infected. **(B)** Levels of RBP-4; **(C)** Levels of CETP; **(D)** Levels of Lp-PLA2; **(E)** Levels of OGT; **(F)** Levels of GLUT-1; **(G)** Levels of LDH-A. Values were normalized to the mock-infected control in each graph.

On the 9th day post-inoculation, tissues were collected from three random pigs in each group to analyze the levers of metabolism. The results demonstrated that APOE expression increased in the kidney following ASFV infection but significantly increased in other tissues (Figure 2A). This observation may be linked to lipid accumulation in the kidney resulting from disturbances in lipid metabolism. Following ASFV infection, RBP-4 expression decreased in the liver but exhibited a significant increase in other tissues (Figure 2B), potentially due to the high expression of RBP-4 in healthy liver tissue. Lp-PLA2 expression levels decreased in various tissues, whereas CETP expression significantly increased across all tissues (Figures 2C,D). In the serum of the experimental group, OGT expression significantly decreased, whereas GLUT-1 expression markedly increased (Figures 2E,F), indicating that disruptions in glucose metabolism contributed to elevated blood glucose levels. Additionally, LDH-A expression was significantly upregulated in the spleen, kidneys, and mesenteric lymph nodes (Figure 2G), suggesting that disruptions in glucose metabolism were primarily observed in these tissues.

### 3.3 ASFV infection promotes lipid synthesis in WSL cells

**Figure 3:**
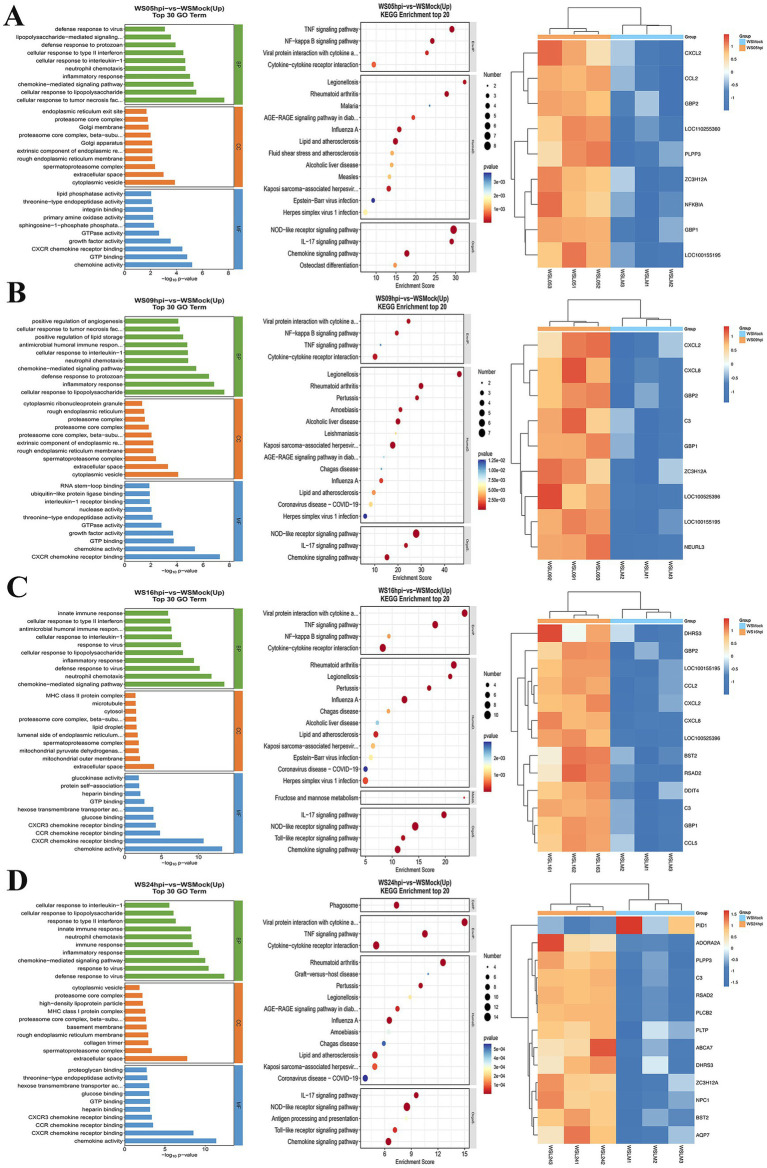
Transcriptomic analysis of ASFV-infected WSL cells. WSL cells were infected with ASFV and total RNA were prepared at 5 **(A)**, 9 **(B)**, 16 **(C)**, and 24 **(D)** hpi for transcriptomic analysis. The data were analyzed using GO, KEGG, and heatmap.

To assess whether ASFV infection influences lipid synthesis, WSL cell samples were collected at different time points post-infection and analyzed using transcriptomics. GO and KEGG analyses revealed a gradual increase in the number of DEGs as infection progressed. The enrichment of lipid synthesis-related pathways was observed at various time points during ASFV infection (Figure 3). These alterations are associated with ASFV entry into host cells and the modulation of the host response. Heatmap analysis of the DEGs at multiple time points post-infection revealed a consistent increase in the expression of partial lipid synthesis-related genes as the infection progressed (Figures 3C,D). Notably, PID1 is a gene closely associated with the regulation of lipid metabolism and was first shown to be significantly downregulated 24 h after ASFV infection (Figure 3D). Conversely, other genes that promote lipid synthesis were upregulated at various time points following infection.

### 3.4 Validation of ASFV-induced lipid synthesis *in vitro*

**Figure 4:**
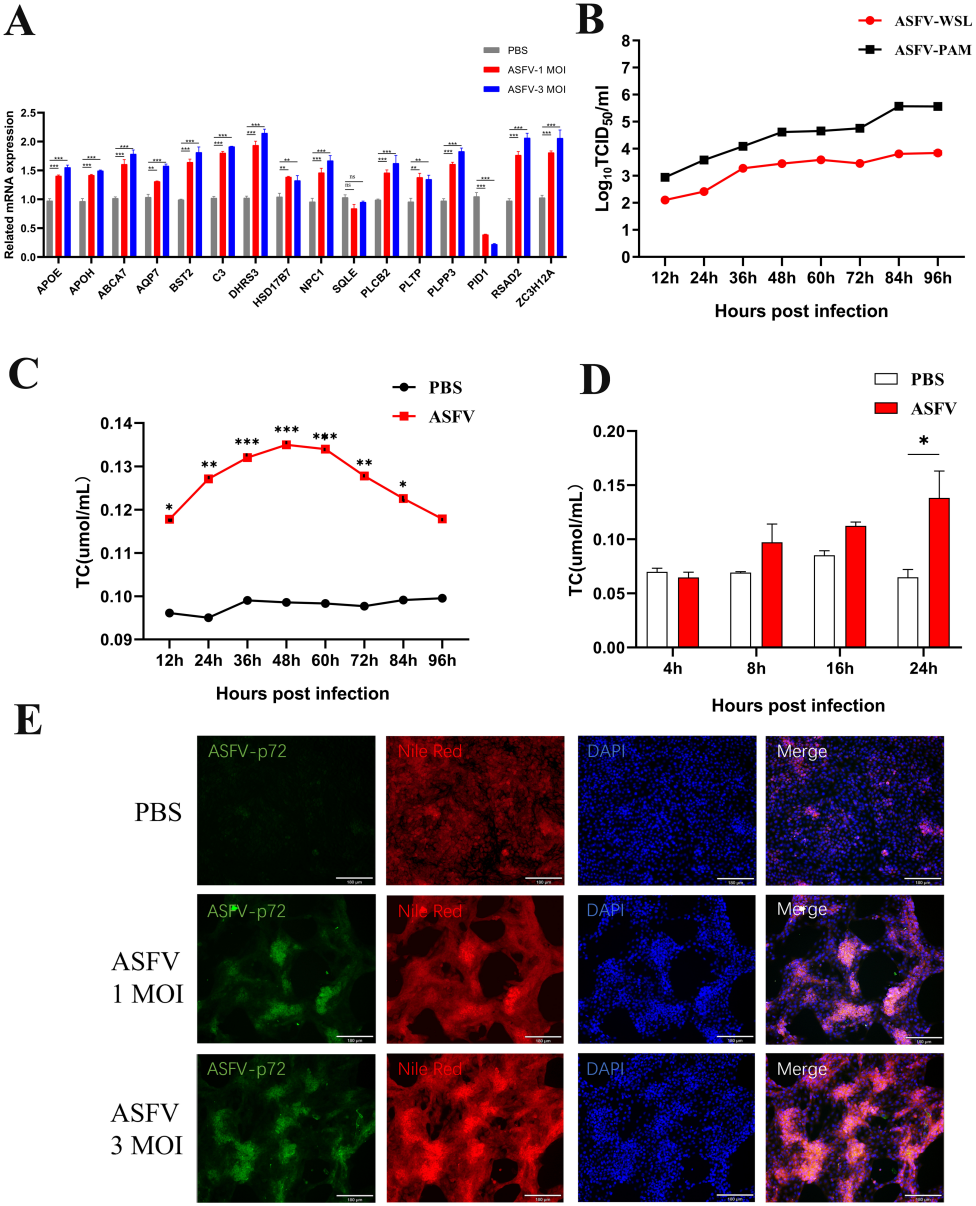
Validation of ASFV-induced lipid synthesis *in vitro*. **(A)** The expression of genes was confirmed by RT-qPCR. ASFV was inoculated into WSL cells and the total RNA was prepared at 24 hpi and detected by RT-qPCR. **(B)** ASFV growth curves were analyzed in both WSL and PAM cells. **(C,D)** Intracellular cholesterol increased after ASFV infection. ASFV was inoculated into WSL cells and the cellular lysate were collected at 12, 24, 36, 48, 72, 96 hpi. Intracellular cholesterol levels were measured by CheKine™ Micro Total Cholesterol (TC) Assay Kit. **(E)** Nile red staining was used to confirm that ASFV infection increases intracellular cholesterol levels in WSL cells. Magnification, х200.

WSL cells were infected with ASFV (MOI 1 or MOI 3) and total RNA was collected 24 h post-infection. RT-qPCR was used to analyze the levels of lipid metabolism. The results indicated that partial lipid metabolism-related genes were upregulated to varying extents in WSL cells infected with different amounts of ASFV. The most significantly differentially expressed genes included PLPP3, DHRS3, ABCA7, BST2, PID1, and ZC3H12A, which are associated with lipid metabolism (Figure 4A), in accordance with the results of the transcriptomic analyses. The growth curve of ASFV on WSL cells was plotted (Figure 4B), and the cells were collected at various time points post-infection to measure intracellular cholesterol levels. A significant increase in cholesterol levels was detected as early as 12 h post-infection, with levels continuing to rise as the infection progressed. The cholesterol levels increased progressively, peaking at 48 h post-infection (Figure 4C), before gradually declining. This decline may be attributed to a decrease in the viability of infected cells. A significant increase in intracellular cholesterol levels was observed only 24 h after ASFV infection in PAM cells (Figure 4D), potentially due to differences in ASFV replication efficiency between the two cell lines. Additionally, Nile red staining at 24 h post-infection in WSL cells revealed that ASFV infection increased cholesterol production in these cells (Figure 4E).

### 3.5 Increased lipid synthesis enhances ASFV replication

**Figure 5:**
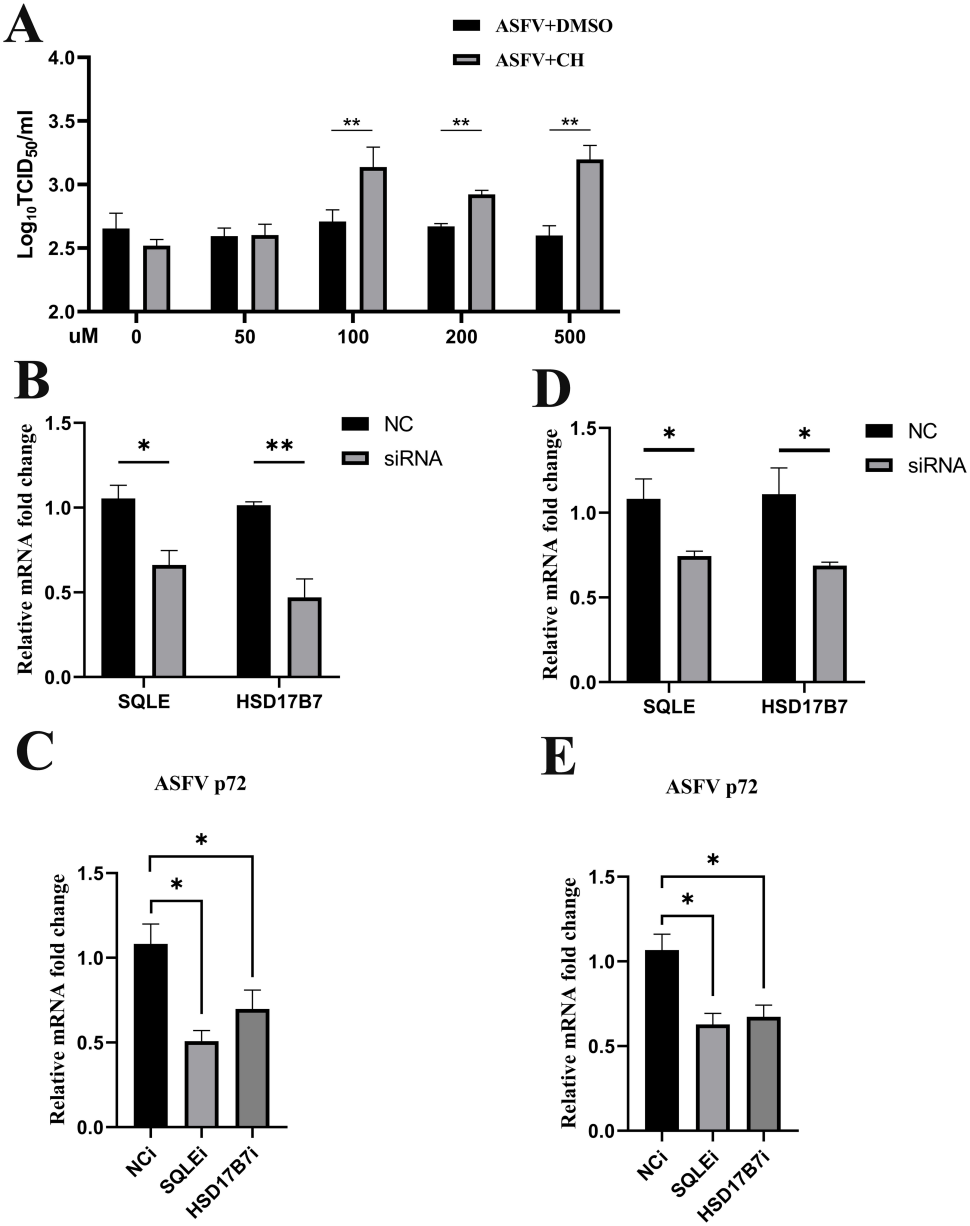
Increased lipid synthesis enhances ASFV replication. **(A)** Cholesterol increased ASFV replication. Cholesterol was added in varying amounts to the cell culture supernatant 2 h after ASFV inoculation to assess its effect on ASFV replication in WSL cells. **(B–E)** Knocking-down SQLE and HSD17B7 decrease ASFV replication. **(B,C)** WSL cells were transfected with interfering RNA for 6 h, followed by ASFV inoculation; **(D,E)** WSL cells were inoculated with ASFV for 6 h, followed by transfection with interfering RNA. The knockdown effects of SQLE and HSD17B7 were observed, and ASFV replication was measured 24 h post-inoculation (NC, negative control; siRNA, siRNA-SQLE/siRNA-HSD17B7).

To assess the influence of lipid synthesis on ASFV replication, various concentrations of small-molecule cholesterol (CH) were added to the cell culture supernatant 2 h post-infection, and replication was evaluated 24 h later. These findings indicated that small-molecule cholesterol significantly increased ASFV replication (Figure 5A). To investigate the effects of lipid synthesis gene downregulation on ASFV replication, the key lipid synthesis regulatory genes *SQLE* and *HSD17B7* were knocked down using small interfering RNA (siRNA) transfection (Figures 5B,C). The results revealed that the suppression of *SQLE* and *HSD17B7* expression, which occurred either 6 h before or after viral infection, led to a significant reduction in viral replication (Figures 5D,E). In summary, the inhibition of lipid synthesis considerably decreased ASFV replication.

## 4 Discussion

The metabolic condition of the host is a vital determinant of viral replication (Albert et al., 2022). Viral-encoded proteins alter host metabolism to meet the demand of the virus (Thaker et al., 2019), demonstrating an active interaction between the virus and the host. The ASFV genome encodes 150 to 200 proteins, many of which are crucial for regulating the host immune system (Cackett et al., 2022; Zheng et al., 2022; Niu et al., 2023). Studies have shown that ASFV infection in porcine alveolar macrophages leads to significant changes in over 90 metabolites, mostly amino acids and TCA cycle intermediates (Xue et al., 2022). Metabolomic analysis of cells infected with ASFV has revealed that ASFV infection facilitates the host’s TCA cycle and amino acid metabolism, increasing ASFV replication (Xue et al., 2022). Here, using *in vivo* experiments, we revealed that severe lipid metabolism disorders occur in organisms during ASFV infection.

Serological examinations of ASFV-infected pigs show notable shifts in lipid metabolism-related cytokines, including HDL-C, LDL-C, TC, TG, Lp-*α*, and Apo-A1. These results suggest that viral infection interferes with lipid synthesis and transportation in tissues and organs. Consequently, lipid metabolism is impacted, leading to lipid accumulation and unusually high cholesterol and other lipid levels. Furthermore, the notable reductions in the levels of GSP, GHb, and glucose in the serum of infected pigs indirectly reflect the influence of viral infection on energy metabolism. Analyses of gene expression related to lipid metabolism in various virus-infected tissues revealed decreased expression of APOE and Lp-PLA2, which could be related to the dysfunction induced by viral infection. The expression of RBP-4 and CETP was elevated, whereas RBP-4 expression was significantly reduced in the liver. This may be increased lipid synthesis caused by viral infection and the body’s regulation of metabolite transport. These findings suggest that the expression of LDH-A and GLUT1 is significantly elevated while OGT expression is reduced, indicating that certain mechanisms that regulate glycolysis are activated while glycolysis is affected. The increased expression of these glucose metabolism-related genes is likely associated with the direct regulation of viral proteins. In summary, the viral regulation of lipid metabolism and glucose metabolism occurred at different times during ASFV infection and in different tissues.

Total RNA was extracted from WSL cells at various times following viral infection *in vitro* for transcriptomic studies. The data indicated that the upregulated expression of lipid metabolism-related genes, such as PLPP3 and ZC3H12A, occurred as early as 5 h post-infection. Additionally, upregulated expression of antiviral genes such as GBP1, GBP2 and CCL2 was also observed, which is closely related to the inflammatory response elicited by viral infection. The upregulated expression of the C3 and ZC3H12A genes and upregulated expression of antiviral genes, such as CXCL8 and NEURL3, occurred at 9 h post-infection. Progressing to 16 h post-infection, the expression of genes related to lipid raft formation, such as BST2 appeared, was upregulated, and the expression of the antiviral gene RSAD2 was upregulated. At 24 h post-infection, many lipid metabolism-related genes, including PLPP3, C3, ABCA7, ZC3H12A, BST2, AQP7, PLTP, and PLCB2, were significantly upregulated. In particular, significant downregulation of genes related to fat deposition, such as PID1 (Wang et al., 2006; Qian et al., 2010), was observed. GO and KEGG analyses revealed significant enhancements in lipid synthesis-related pathways during different phases of ASFV replication. Lipids play crucial roles throughout the virus’s lifecycle and serve as an energy source for viral replication through fatty acid oxidation. These results suggest that different stages of ASFV infection are able to modulate the lipid metabolism of host cells to promote viral self-replication.

Further *in vitro* validation through qPCR was performed, and alterations in the expression of lipid metabolism-related genes mentioned above corresponded with transcriptomic analysis findings at 24 h post-infection in WSL cells. Studies have identified SQLE and HSD17B7 as crucial regulatory genes in lipid synthesis (Ohnesorg et al., 2006; Seth et al., 2006; Zhang et al., 2019; Tan et al., 2020; Chen W. et al., 2023). The subsequent knockdown of these key lipid synthesis genes, SQLE and HSD17B7, significantly reduced ASFV replication in WSL cells. Conversely, the addition of CH to the cell culture medium significantly enhanced ASFV replication. These findings suggest that ASFV proliferation in vitro is strongly dependent on host lipid synthesis and cholesterol flux. In summary, ASFV infection results in notable alterations in the expression of partial metabolism-related genes, predominantly upregulating genes involved in lipid synthesis. Lipid synthesis plays a key role in the invasion and replication of ASFV.

This study is the first to demonstrate the effects of ASFV infection on lipid and glucose metabolism through animal experiments. Further exploration revealed that ASFV augments self-replication by modulating host cell metabolism. However, the specific mechanisms by which viruses regulate host cell metabolism are still being studied. Understanding and targeting viral regulation of host metabolism could alleviate the suppression of host immune responses by viruses, thereby aiding in vaccine development and application. Continued in-depth research into viral regulation of host metabolism is essential for preventing and controlling large DNA viruses.

## 5 Conclusion

This investigation revealed that ASFV infection disrupts both lipid and glucose metabolism and promotes the upregulation of host genes associated with lipid synthesis. High level of cholesterol promoted ASFV replication.

## Data Availability

The raw data supporting the conclusions of this article will be made available by the authors, without undue reservation.

## Footnotes

^1^ https://cloud.oebiotech.cn
